# *Clostridium perfringens* Enterotoxin: The Toxin Forms Highly Cation-Selective Channels in Lipid Bilayers

**DOI:** 10.3390/toxins10090341

**Published:** 2018-08-22

**Authors:** Roland Benz, Michel R. Popoff

**Affiliations:** 1 Department of Life Sciences and Chemistry, Jacobs University, Campusring 1, 28759 Bremen, Germany; r.benz@jacobs-university.de; 2 Bacterial Toxins, Institut Pasteur, 28 rue du Dr Roux, 75015 Paris, France

**Keywords:** pore-forming toxins, *clostridium perfringens* enterotoxin (CPE), propidium iodide uptake, MTT-test, channel formation, cation-selectivity, lipid bilayer membrane

## Abstract

One of the numerous toxins produced by *Clostridium perfringens* is *Clostridium perfringens* enterotoxin (CPE), a polypeptide with a molecular mass of 35.5 kDa exhibiting three different domains. Domain one is responsible for receptor binding, domain two is involved in hexamer formation and domain three has to do with channel formation in membranes. CPE is the major virulence factor of this bacterium and acts on the claudin-receptor containing tight junctions between epithelial cells resulting in various gastrointestinal diseases. The activity of CPE on Vero cells was demonstrated by the entry of propidium iodide (PI) in the cells. The entry of propidium iodide caused by CPE was well correlated with the loss of cell viability monitored by the 3-(4,5-dimethylthiazol-2-yl)-2,5-diphenyltetrazolium bromide (MTT) test. CPE formed ion-permeable channels in artificial lipid bilayer membranes with a single-channel conductance of 620 pS in 1 M KCl. The single-channel conductance was not a linear function of the bulk aqueous salt concentration indicating that point-negative charges at the CPE channel controlled ion transport. This resulted in the high cation selectivity of the CPE channels, which suggested that anions are presumably not permeable through the CPE channels. The possible role of cation transport by CPE channels in disease caused by *C. perfringens* is discussed.

## 1. Introduction

*Clostridium perfringens* is a ubiquitous spore-forming anaerobic bacterium which is responsible for various diseases in humans and animals including gangrene, and gastro-intestinal diseases (food poisoning diarrhea, enteritis, necrotic enteritis, and enterotoxemia). *C. perfringens* synthesizes numerous toxins and the strains are divided into five toxinotypes (A to E) according to the production of four toxins (alpha, beta, epsilon, and iota) [1]. *C. perfringens* enterotoxin (CPE) is the causative toxin of food poisoning diarrhea and enteritis in humans and more rarely for enteritis in animals. Only a few number of *C. perfringens* strains (about 5%) contain the *cpe* gene and produce CPE. The *cpe* gene is mainly harbored by *C. perfringens* type A now referred to as *C. perfringens* type F, and more occasionally by *C. perfringens* types C and D [2,3]. Most of *C. perfringens* type E possess a silent *cpe*, but some strains express a functional variant CPE [4,5]. CPE sequence is almost identical in all strains except in *C. perfringens* type E containing a variant CPE [2]. In most *C. perfringens* type A strains (about 70%) involved in food poisoning, *cpe* is located on the chromosome flanked by insertion sequences (IS) IS*1469* and IS*1470*, whereas in other type A strains of food-borne poisoning (about 30%) and non-food-borne gastrointestinal disease *cpe* is on the plasmid of pCPF5603 or pCPF4969 families [2,3].

In contrast to the other *C. perfringens* toxins, CPE is only produced during sporulation which occurs in special culture media and in the intestine. Thus, in food poisoning, ingestion of a large number of *C. perfringens* leads to intestinal proliferation, sporulation, CPE production and release in the intestinal content [2,6]. Via its C-terminal domain, CPE binds to “some” claudins, which are four-transmembrane domain proteins. Claudins are major components of tight junctions between epithelial or endothelial cells and have a critical role in the barrier function [7,8,9,10]. However, CPE only recognizes some isoforms among the 27 members of the claudin family. Claudins 3, 4, 6, 7 and 9 are high affinity CPE receptors, whereas claudins 1, 2, 8, 14, and 19 are low or medium affinity CPE receptors [2,11,12,13]). Interestingly, claudins 3 and 7 are among the most highly expressed claudins in the intestine, while claudin 4 is expressed in proximal gastric glands, the lateral membrane of the villus surface epithelial cells in both the small intestine and colon but also in kidney and lung [7,14,15]. Claudins control epithelial barrier permeability. Notably, claudin 7 predominantly increases permeability to anions, while claudins 3, 6, 9 decrease permeability to anions [7]. CPE binds through its C-terminal domain (amino acids 184–319) to both claudin extracellular loops resulting in the formation of a 90 kDa small complex which subsequently oligomerizes and associates to other non-receptor claudins to form large ~450 kDa complexes called CH-1. Larger complexes (~650 kDa, CH-2) contain, in addition, another tight junction protein occludin. CH-1 complexes including 6 CPE molecules induce functional pores, whereas CH-2 activity is unclear [2,16,17,18,19,20,21]. Low doses of CPE induce limited Ca^++^ influx and apoptosis by activation of the classical caspase 3/7 pathway, and high CPE doses cause massive Ca^++^ influx and cell death by oncosis [2,16,18,22]. This results in the removal of claudins from the cell membrane and subsequent degradation. Tight junctions disintegrate showing a decreased number of strands and loss of the strand network complexity leading to increased permeability of epithelial cell monolayers or small intestine and colon barrier notably by increasing the paracellular permeability [15,16,23,24,25]. Binding of CPE C-terminal domain to claudins induce conformational change of claudin extracellular loops, resulting in the disruption of lateral claudin interjunctions [26].

CPE shows the characteristic structure of a β-pore-forming toxin. Notably, the N-terminal domain is structurally homologous to that of the aerolysin β-pore-forming toxin family and contains an α-helix and β-strand (residues 81–106) able to undergo a conformational change in amphipathic β-hairpin with alternate hydrophobic/hydrophilic amino acids. Upon CPE oligomerization, the amphipathic β-hairpins can assemble in membrane inserting β-barrel [27,28]. Deletion or mutations of the 81–106 sequence yield CPE variants unable to form pores and which are non-cytotoxic [29,30]. CPE at a high concentration has been found to induce small pores in artificial membranes from synthetic phosphatidylethanolamine and phosphatidylserine or in asolectin bilayers [31,32], and cationic pores in CaCo-2 cells [33]. We further characterized the CPE pore-forming activity in synthetic lipid bilayer constituted of phosphatidylcholine that is more representative of a plasma cell membrane and allows better comparison with other pore-forming toxins investigated in these same conditions.

Here we show that CPE forms highly cation-selective channels in planar lipid membranes formed of the zwitterionic diphytanoyl phosphatidylcholine. The cation selectivity is caused by a hot-spot of negative charges discussed previously in several studies, which results in dependence of channel conductance on the square root of the bulk aqueous salt concentration [34,35,36]. The fit of the single channel conductance as a function of the bulk aqueous concentration allows also a rough estimate of the CPE channel size, which was found to have a diameter of about 1.4 nm.

## 2. Results

### 2.1. CPE Pore-Forming Activity on Vero Cells

**Figure 1:**
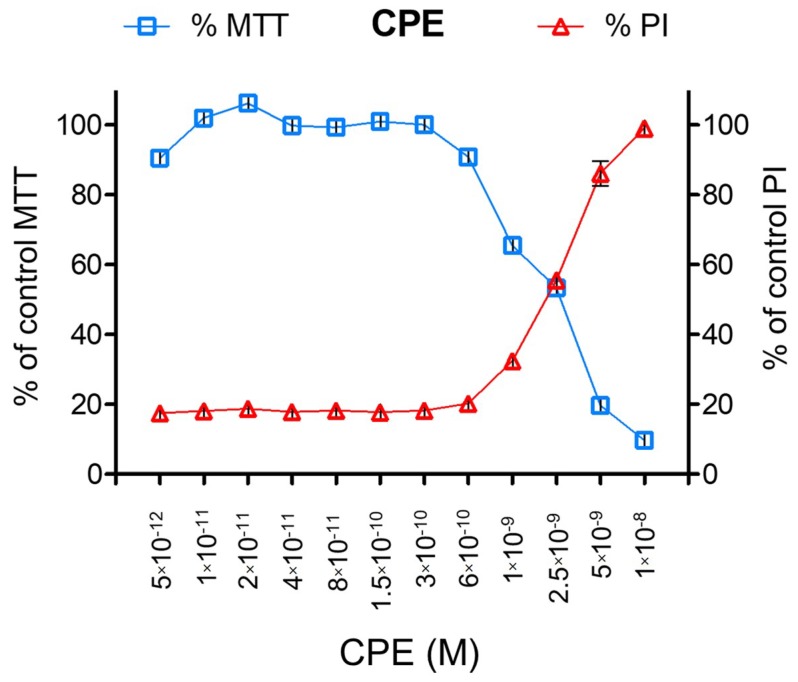
CPE activity on Vero cells monitored by cell entry of propidium iodide and cell viability with MTT test. Vero cells grown on 96-well plate were exposed to GST-CPE serial dilutions and incubated at 37 °C for 2.5 h. Entry of propidium iodide was monitored by spectrofluorimetry and cell viability by the MTT test.

CPE was produced as a recombinant protein with GST-tag (Figure S1). CPE activity on Vero cells was checked by monitoring the entry of propidium iodide (PI) as already tested with the mouse carcinoma cell line FM3a [37] or with other pore-forming toxins such as *C. perfringens* epsilon toxin (ETX) which form small pores in the plasma membrane [38]. As shown in Figure 1, 2.5 nM CPE induced 50% PI entry within 2.5 h incubation, which correlated with 50% loss of cell viability monitored by the MTT test. This further supports that CPE induces pores in Vero cell’s plasma membrane mediating PI passage and that pore formation is associated with the loss of cell viability.

To check the potential influence of the GST-tag on CPE activity. Thrombin-cleaved and uncleaved CPE were compared for their activity on Vero cells. No significant difference was observed in PI cell entry and cell viability between cleaved and uncleaved CPE (Figure S2).

### 2.2. Pore-Forming Activity of CPE in Lipid Bilayer Membrane

**Figure 2:**
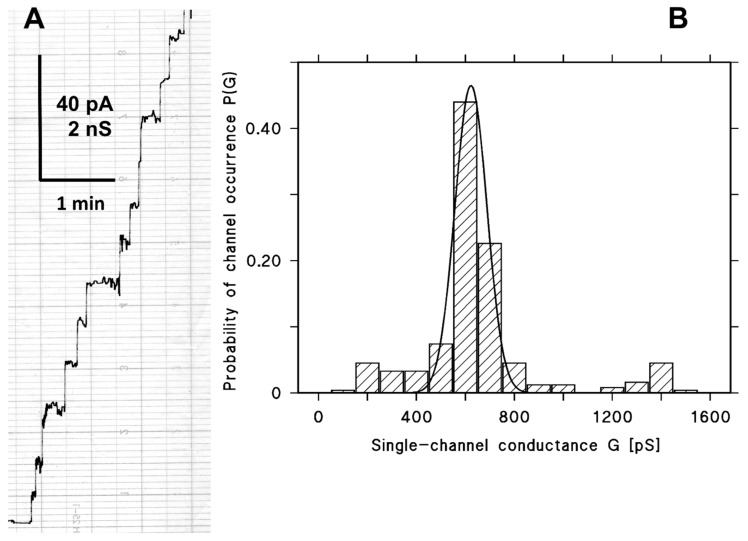
Results of single-channel measurements with *C. perfringens* enterotoxin (CPE). (**A**) Single-channel recording of PC/n-decane membranes after the addition of about 3.5 ng/mL cleaved CPE to one side of the black lipid bilayer. The addition of CPE resulted in rapid occurrence of single current steps with a long lifetime. The applied voltage was 20 mV and the temperature was 20 °C; (**B**) histogram of the channels formed by cleaved CPE in PC/n-decane lipid bilayer membranes. The solid line represents a fit of the histogram with a Gaussian distribution. The maximum of the distribution is at a probability of 0.46 ± 0.02 and the conductance is 623 ± 65 pS for in total 243 single events taken from 12 individual membranes, V_m_ = 20 mV, T = 20 °C.

After the formation of a diphytanoyl phosphatidylcholine/n-decane membrane in a 1 M KCl-solution, cleaved CPE (1.74 μg/mL, 50 nM) was added to one side of the membrane, the cis-side of the membrane in a small amount (about 3.5 ng/mL). After about 2–3 min, presumably caused by slow aqueous diffusion of the toxin through the unstirred layer near the membrane surface, the membrane conductance increased in a stepwise fashion (Figure 2A), indicating the formation of ion-permeable channels in the membrane. The current increase was very rapid, as Figure 2A indicates, and the membrane conductance increased subsequently by more than three orders of magnitude within about 30 min after the addition of cleaved CPE. Figure 2B shows a histogram of the distribution of channels formed by cleaved CPE. Most channels (about 70%) had a conductance of 500 and 600 pS and only a small fraction of channels had a much higher conductance presumably caused by the fact that single channels could not be separated on the time scale of our detection system because of the rapid conductance increase. This result indicated that the cleaved CPE channels were quite homogeneous. The solid line in Figure 2B shows a Gaussian fit of the probability for the occurrence of certain conductance steps. The maximum of the distribution is at a probability of 46 ± 2% and the conductance was 623 ± 65 pS for in total more than 200 single events obtained from 12 individual membranes.

### 2.3. Single-Channel Conductance of CPE Channel

To study the properties of the cleaved CPE channels in more detail, we performed single-channel experiments at different KCl-concentrations. In addition, we also studied the effect of different anions and cations of CPE channel conductance. The results are summarized in Table 1. Interestingly, the average single-channel conductance, G, did not increase with a linear function for increasing KCl-concentrations in the aqueous phase (Table 1), which would be expected for a wide water-filled channel similar to those formed by general diffusion porins of Gram-negative bacteria [39]. Instead, the conductance increased as a function of the square root of the bulk aqueous salt concentration, which indicates the influence of net charges on channel conductance [35] (see Discussion). Table 1 shows also the results for single channel measurements of cleaved CPE in 1 M KCH_3_COO^−^ and in 1 M LiCl. Whereas the conductance of the CPE channels in 1 M LiCl was considerably below that in 1 M KCl, the conductance in 1 M KCH_3_COO^−^ was almost identical to that in 1 M KCl. This result suggests that the CPE channel is highly cation selective and that the permeation of anions through the channel is relatively low and could even be negligible.

### 2.4. Selectivity of the CPE Channel

Further information about the structure of the cleaved CPE channel and its influence on ion transport was obtained from zero-current membrane potential measurements in the presence of fivefold salt gradients (100 mM versus 500 mM KCl, LiCl or potassium acetate). The salt gradients were established across lipid bilayer membranes in which a considerable number (about 100) of CPE channels were reconstituted. In the case of KCl, the gradient resulted in an asymmetry potential of 33.3 (±2.4) mV (mean of 3 measurements) at the more dilute side of the membrane for KCl. This result indicated preferential movement of potassium ions over chloride through the channel at nearly neutral pH. The zero-current membrane potentials were analyzed using the Goldman-Hodgkin-Katz equation [40]. The ratio of the potassium permeability, P_K_, divided by the chloride permeability, P_Cl_, was about 11, indicating indeed a high cation selectivity of the CPE channel. A similarly high cation selectivity was measured for LiCl and potassium acetate. In these cases, we observed 5-fold gradient asymmetry potentials of 29.7 and 34.3 mV at the more dilute side, respectively (see Table 2). The corresponding ratios of cation permeability P_cation_ divided by anion permeability P_anion_ were 8.3 for LiCl and 13 for potassium acetate. Although size and/or mobility of cations and anions seemed to influence somewhat the selectivity of the cleaved CPE channel, it is quite clear that anions have an extremely low permeability if any through the cleaved CPE channel, which is in agreement with the single channel data.

### 2.5. Voltage Dependence of the CPE Channel

**Figure 3:**
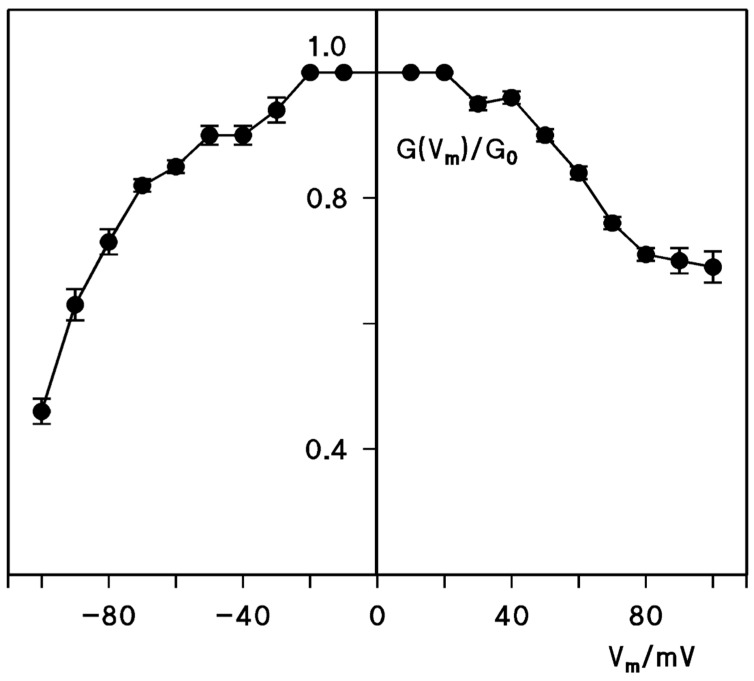
Voltage dependence of *C. perfringens* enterotoxin (CPE) when the protein was added to one side of the membrane. The ratio of the conductance (G) at an applied voltage (V), divided by the initial value of the conductance (G_0_), immediately after the turning on of the voltage. The aqueous phase contained 1 M KCl and about 50 to 100 ng/mL CPE added to the cis-sides of the membranes. The membranes were formed from diphytanoyl phosphatidylcholine/*n*-decane at a temperature of 20 °C. The applied voltage refers to the cis-side of the membranes. The full points show the mean values (±SD) of three individual membranes.

Channels formed by membrane-damaging toxins or by B-components (binding components) of A-B type of toxins form often voltage-dependent channels that are open under physiological conditions, i.e., when the voltage on the trans-side of toxin or binding-component addition has a negative sign [41,42]. This results in open toxin channels and leads to dissipation of ionic and other gradients across the cell membrane, which is followed by membrane rupture and cell death. To test if a similar asymmetric voltage-dependence is given for cleaved CPE channels, we performed multi-channel experiments where cleaved CPE was only added to one side of the lipid bilayer membranes, the cis-side. After insertion of a large number of cleaved CPE channels, increasing voltages with positive and negative signs were applied to the membranes. The conductance at given positive and negative voltages were divided by the conductance at ±10 mV. The results were plotted as a function of the voltage applied to the cis-side. Figure 3 shows the mean (±SD) of three experiments of this type. Compared to other toxins, CPE shows similar asymmetric current-voltage curve indicating oriented insertion of the cleaved CPE channel into the membranes. For voltages positive at the cis-side (corresponding to negative voltages at the trans-side, the physiological condition), the conductance decreased by about 30% at 90 mV, whereas it decreased by almost 60% at −90 mV.

## 3. Discussion

### 3.1. CPE Has a High Pore-Forming Capacity

The addition of small amounts of CPE on the order of 10 to 100 ng/mL to one or both sides of artificial lipid bilayer membranes resulted in the formation of many small ion-permeable channels. Without any problem, it was possible to reconstitute more than 10^5^ to 10^6^ CPE channels in a lipid bilayer with a surface area of about 0.4 mm^2^. Our results suggest that most of the channels were homogeneous and had a single channel conductance of about 600 pS in 1 M KCl. Channels formed in the lipid bilayer membranes without the presence of any receptor protein, which are different claudins in the case of CPE [26]. It is noteworthy that this is typical for the interaction of a variety of toxins with artificial lipid bilayer membranes [38,41,42]. Whereas the absence of receptors makes cell lines resistant for the attack of toxins, lipid bilayers with their smooth surfaces are excellent targets for the interaction with toxins [43,44].

### 3.2. The Channel Formed by CPE C. perfringens Is Cation Selective

**Figure 4:**
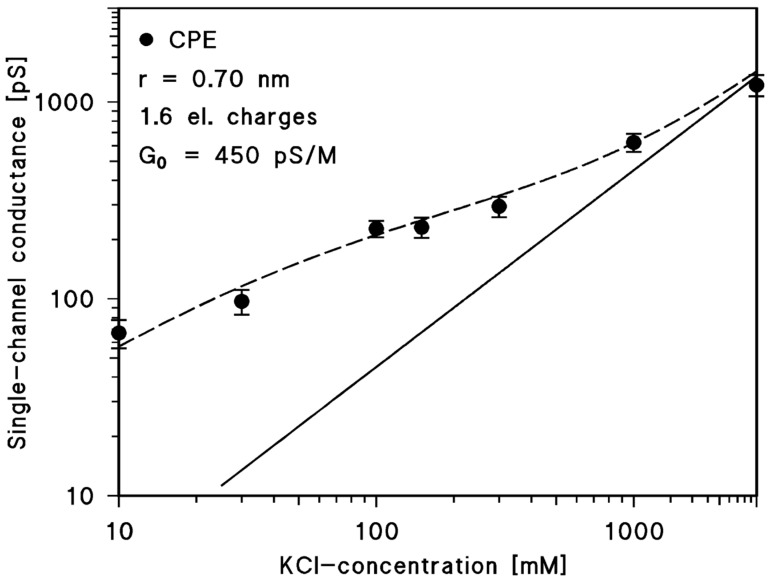
Single-channel conductance of the CPE channel of *C. perfringens* as a function of the KCl concentration in the aqueous phase. The broken line represents the fit of the single-channel conductance data (filled circles) with Equations (1)−(3), assuming the presence of negative point charges (1.6 negative charges; *q* = −2.56 × 10^–19^ As) at the channel mouth and assuming a channel diameter of 1.4 nm. c, concentration of the KCl solution in mM (millimolar); Average single-channel conductance in pS (picoSiemens, 10^−12^ S). The solid (straight) line shows the single-channel conductance of the cell wall channel that would be expected without point charges assuming a single-channel conductance of 450 pS/M. It corresponds to a linear graph between channel conductance and bulk aqueous concentration at a double logarithmic scale using Equation (4) and the salt concentration of the bulk aqueous phase.

Single-channel and ions selectivity measurements revealed that CPE formed cation-selective channels, which has also been noticed in a previous study, although the conductance measured here in 100 mM KCl (227 ± 22 pS) differed considerably to the earlier data (565 ± 15 pS) under the same conditions [31]. A look at the dependence of single-channel conductance on aqueous salt concentration suggested that it was dependent on the square root of the salt (cation) concentration (see Table 1). A comparison with previous studies of channel properties suggested that a hot-spot of negative charges in or near the channel could be responsible for this type of dependence of conductance on ion strength in the aqueous phase [35,36,45]. Equations (1)–(4) describe the effect of negative charges on the single channel conductance following an earlier published formalism [35,46]. A best fit of the dependence of channel conductance on ionic strength given in Table 1 was obtained by using the theoretical treatment described in the Materials and Methods section and assuming that 1.6 negatively charged groups (*q* = −2.56 × 10^–19^ As) are located at the pore mouth and that its radius is approximately 0.70 nm. Figure 4 demonstrates that a reasonable fit of the experimental data is possible for these parameters. Similarly, it is clear from Figure 4 that the influence of the hot-spot of negative charges is rather small on channel conductance at high ionic strength. Correction of the single-channel conductance for the presence of point charges at the channel mouth resulted in a linear graph of conductance as a function on aqueous salt concentration on a double logarithmic scale (solid line in Figure 4). Similar considerations apply also to the discussion of the zero-current membrane potentials, which means that because of the negative charges at the channel only part of the full bulk aqueous salt gradient drops across the channel, i.e., the zero-current membrane potential could be higher if the full cation gradient dropped across the channel, indicating that the anion could not permeate through the CPE channel.

### 3.3. Comparison with Other Pore-Forming Toxin (PFT) Channels

The high cation selectivity of the CPE channel agrees very well with the putative structure of the CPE channel. The 3D-structure of water-soluble CPE has been derived from X-ray crystallization studies [27,28]. It shows a striking homology to the structure of aerolysin and *C. perfringens* ε-toxin, which show the same three domains [27,28,43,47,48]. This family is characterized by an α-helix and β-strand (residues 81–106) able to undergo a conformational change in an amphipathic β-hairpin with alternate hydrophobic/hydrophilic amino acids. The pore-forming β-hairpin motif lining the CPE channel is hidden within domain II, similar to the situation of the other two pore-forming toxins [28]. Site-directed mutagenesis of some of the amino acid residues 81–106 within domain II of CPE suggested indeed that they are part of the β-hairpin motif [28,30]. Assuming this is correct, the pore-forming domain of CPE contains three negatively-charged glutamic acids (E80, E94 and E101) and no positively charged amino acid per CPE monomer. Assuming that the channel is formed by an oligomer, for example by a heptamer, it is clear that anions cannot permeate the channel because of the many negative charges within the channel. Aerolysin and ε-toxin contain, in contrast to this, an excess of positively charged amino acids (Four lysines and three glutamates for aerolysin; one lysine and no negative charge for ε-toxin), which makes the corresponding channels anion selective [28,49,50]. Charges within the channel-forming domain definitely control the flux of ions through the channel.

Table 3 shows a comparison of the channel characteristics of different toxins that are all characterized by the formation of β-hairpins in the oligomeric channels. Aerolysin and α-toxin form heptamers [47,48,51]. It is possible that also ε-toxin forms heptamers [47,52]. All these toxins forming β-barrel cylinders in the membrane have a similar conductance as the CPE channel, which means presumably that they all have similar structures and probably also have a similar size. Only *C. septicum* alpha-toxin has a conductance that is approximately twice that of the other four toxins (see Table 3). It also forms oligomers with an apparent molecular mass of considerably higher than 250 kDa, but it is not known whether it forms heptamers or octamers [53,54]. Assuming a similar structure and the higher conductance it could well be that *C. septicum* alpha-toxin forms octamers. From the channel-forming toxins of Table 3, only CPE forms cation-selective channels, which is well understood if the amino acids lining up the β-hairpins of the seven monomers forming the β-barrel cylinder are considered. All the β-barrel cylinders formed by the other four toxins contain an excess of positively charged groups that makes the channels anion-selective.

Plasma membranes of all eukaryotic cells contain a common phospholipid bilayer backbone and variable sets of proteins and glycoproteins inserted into the lipid bilayer. Bacterial toxins do not have direct access to the phospholipid bilayer since the cumbersome multiple compounds at their surface and requires a specific receptor (protein, glycoprotein, or glycolipid) to interact with cell membranes. Indeed, as discussed, CPE interacts with cell membranes containing specific claudins. Receptors likely trap the toxin molecules toward the lipid bilayer. However, bacterial toxins can directly interact with the synthetic phospholipid bilayer containing no additional compounds. This model of synthetic lipid bilayer allows us to precisely define the characteristics and mechanism of toxin interaction with the lipid bilayer independently of the presence of other membrane compounds. Albeit CPE only interacts with cell membranes containing specific receptors, it is able to form functional pores in the synthetic phospholipid bilayer. We assume that this represents the final step of CPE interaction with the cell membrane. The structure of CPE oligomers and pores is not yet known. Based on the homology with aerolysin, CPE might form an heptameric prepore that has a mushroom shape. Following interaction with the lipid membrane via the cap of the aerolysin prepore, the stalk collapses, extending the β-barrel in the opposite direction, which inserts into the lipid bilayer [55]. However, since CPE forms different complex sizes by association with claudins and other membrane proteins such as occludin [2,16,17,18,19,20,21], the structure of CPE pores on the cell membrane might be more complex than that of the aerolysin pore. The structure of CPE oligomers remains to be defined. CPE is responsible for *C. perfringens* food intoxication which is characterized by diarrhea. CPE pores on intestinal epithelial cells likely contribute to the fluid and electrolyte losses in addition to the disruption of intercellular junctions by disorganization of claudins. Moreover, CPE pore formation induces intestinal cell death by necrosis or apoptosis upon a yet undefined mechanism which further increases the intestinal permeability. Thereby, pore formation is a critical mechanism of CPE activity.

## 4. Materials and Methods 

### 4.1. Materials 

The *cpe* open reading frame was PCR amplified from *C. perfringens* strain 8-6 with the primers P843 5′-ATGGATCCATGCTTAGTAACAATTTAAATCC-3′ and P979 5′-TGAATTCTTAAAATTTTTGAAATAATATTGAATAAGG-3′ adding *Bam*HI-*Eco*RI and cloned into pGEX2T.

CPE was purified as previously described [56]. 3-(4,5-dimethylthiazol-2-yl)-2,5-diphenyltetrazolium bromide (MTT), propidium iodide (PI) were from Sigma (Paris, France). Cleavage by thrombin was performed with the Novagen thrombin kit (69022-3, Novagen Merck, Darmstadt, Germany) according to the manufacturer’s recommendations.

### 4.2. Lipid Bilayer Experiments

The methods used for the lipid bilayer measurements have been described previously in detail [57] from a 1% solution of diphytanoyl phosphatidylcholine (Avanti Polar Lipids, Alabaster, AL, USA) in n-decane. The instrumentation consisted of a Teflon chamber with two aqueous compartments connected by a small circular hole with a surface area of about 0.4 mm^2^. The aqueous salt solutions (Merck, Darmstadt, Germany) were used unbuffered and had a pH around 6, if not indicated otherwise. Native and recombinant *C. perfringens* enterotoxin (CPE) were added from concentrated stock solutions to the aqueous phase bathing membranes in the black state. The temperature was kept at 20 °C throughout. The electrophysiological experiments were performed using a pair of Ag/AgCl electrodes with salt bridges switched in a series with a voltage source and a highly sensitive current amplifier. The amplified signal was monitored with a storage oscilloscope and the reconstitution of CPE channels in the black lipid membrane was recorded with a strip chart recorder. All salts were analytical grade and the temperature was maintained at 20 °C during all experiments. Zero current membrane potential measurements were obtained by establishing a five-fold salt gradient across membranes containing about 100 CPE channels, as described elsewhere [40].

### 4.3. Effect of Negatively Charged Groups Attached to the Channel Mouth

Negative charges at the pore mouth result in substantial ionic strength-dependent surface potentials at the pore mouth that attract cations and repel anions. Accordingly, they influence both single-channel conductance and zero-current membrane potential. The effect of point charges on the single-channel conductance may be calculated according to the treatment of Nelson and McQuarrie [46], which describes the effect of a negative point charge, *q* (in As), on the surface of a membrane. We assume here that the point charge is localized at the channel opening [35]. There, the negative charge causes a potential on the mouth of a channel with a radius *r*, and a total charge *q*, given by:

(1) $$\;\Phi =\frac{2q\cdot e^{-\frac{r}{l_{D}\;}}}{4\pi \cdot \varepsilon_{0}\cdot \varepsilon \cdot r}$$

$\varepsilon_{0}$ (=8.85 × 10^−12^ F/m) and ε (=80) are the absolute dielectric constant of vacuum and the relative constant of water, respectively, and *l_D_* is the so called Debeye length, which controls the decay of the potential (and of the accumulated positively charged ions) in the aqueous phase:

(2) $$\;l_{D}^{2}=\frac{\varepsilon \cdot \varepsilon_{0}\cdot R\cdot T\;}{2\cdot F^{2}\cdot c}$$

*c* is the bulk aqueous salt concentration, and *R*, *T* and *F* have the usual meaning (*RT/F* = 25.2 mV at 20 °C). The concentration of the monovalent cations near the point charge increases because of the negative potential. Their concentration, $c_{0}^{+}$ at the channel mouth is given by:

(3) $$\;c_{0}^{+}=c_{0}\cdot e^{-\frac{\Phi \cdot F\;}{R\cdot T}}$$

The cation concentration, $c_{0}^{+}$, at the mouth of the pore can now be used for the calculation of the effective conductance-concentration curve:

(4) $$\;G(c)\;=G_{0}\cdot c_{0}^{+}$$

$G_{0}$ is the concentration independent conductance of the channel.

### 4.4. Cell Culture

Vero cells were grown in DMEM supplemented with 10% fetal calf serum at 37 °C in a 5% CO_2_ incubator. For cytotoxicity and propidium iodide (PI) assays, Vero cells were grown to confluency in 96-well plates. The monolayers were washed once in DMEM and incubated in PBS containing 5 mM glucose, 0.1% BSA and serial dilutions of CPE in DMEM (100 μL final volume in each well). The viability test using MTT was performed as described previously [58].

### 4.5. Propidium Iodide Influx

For assay of PI entry, Vero cells were grown on 96-well plates until confluency. PI (5 μg/mL) was added in the culture medium together with CPE. At the indicated times, the plates were read with a spectrofluorimeter (Fluoroskan II) (excitation 540 nm and emission 620 nm). The results were expressed as the percentage of fluorescence obtained by treatment with Triton X100 (0.2%) for 30 min at 37 °C.

## Figures and Tables

**Table 1:** Average single-channel conductance of *C. perfringens* enterotoxin (CPE) in different electrolyte solutions ^a^.

Electrolyte	Concentration (M)	G (pS)	N
KCL	0.01	67 ± 11	113
	0.03	97 ± 14	94
	0.1	227 ± 22	118
	0.15	231 ± 27	154
	0.3	295 ± 35	149
	1.0	623 ± 65	243
	3.0	1220 ± 150	261
LiCl	1.0	211 ± 22	96
KCH_3_COO (pH 7)	1.0	619 ± 71	162

^a^ The membranes were formed from diphytanoyl phosphatidylcholine/in *n*-decane. The single-channel conductance was measured at 20 mV applied voltage and T = 20 °C. The aqueous solutions were used unbuffered and had a pH of about 6, if not indicated otherwise. The concentration of CPE was between 3.5 and 20 ng/mL. The average single-channel conductance, G (±SD), was calculated from at least 90 single events. N is the number of events used for the calculation of the average single-channel conductance.

**Table 2:** Zero-current membrane potentials, V_m_, of PC/*n*-decane membranes in the presence of *C. perfringens* enterotoxin (CPE) measured for 5-fold gradients of different salts ^a^.

Electrolyte	V_m_ (mV)	Permeability Ratios P_cation_/P_anion_
KCl	33.3 ± 2.4	11.4
LiCl	29.7 ± 1.7	8.3
KCH_3_COO (pH 7)	34.3 ± 2.3	13.0

^a^ V_m_ is defined as the difference between the potential at the dilute side (100 mM) minus the potential at the concentrated side (500 mM). The aqueous salt solutions were used buffered and had a pH of 6, if not indicated otherwise; T = 20 °C. CPE was added to the cis-side of the membranes in a concentration of about 50 ng/mL. The permeability ratio P_cation_/P_anion_ was calculated from the zero-current potentials using the Goldman-Hodgkin-Katz equation [40]. The zero-current potentials are given as the mean (±SD) of at least three individual experiments.

**Table 3:** Comparison of the channel properties of *C. perfringens* enterotoxin (CPE) with those of aerolysin of *Aeromonas sobria*, alpha-toxin of *Staphylococcus aureus*, epsilon-toxin of *C. perfringens* and *Clostridium septicum* alpha-toxin.

Toxin	Single-Channel Conductance in 1 M KCl	Zero-Current Membrane Potential for 5-Fold KCl Gradients	Permeability Ratio P_K_/P_Cl_
	G [pS]	V_m_ [mV]	P_K_/P_Cl_
CPE	623	+33	11.4
Aerolysin	650	−24	0.21
*S. aureus* α-toxin	820	−22	0.25
ε-toxin	550	−19	0.30
*C. septicum* alpha-toxin	1250	−15	0.38

V_m_ is the electrical potential on the dilute side minus the potential of the concentrated side for a five-fold KCl-gradient across the membranes. The membranes were formed from diphytanoyl phosphatidylcholine/*n*-decane. The aqueous salt solutions were unbuffered and had a pH value of about 6. The permeability ratio, P_K_/P_Cl_, was calculated using the Goldman-Hodgkin-Katz equation [40]. The results for epsilon-toxin were taken from Ref. [38] and those for aerolysin and α-toxin were taken from Ref. [50]. The results for *C. septicum* alpha-toxin were taken from Reference [54].

## Supplementary Materials

The following are available online at http://www.mdpi.com/2072-6651/10/9/341/s1, Figure S1: SDS-PAGE of GST-CPE and CPE. Lane a: GST-CPE, 3 μg; lane b, CPE (3 μg) after treatment of GST-CPE with biotinylated thrombin and removing by streptavidin agarose beads. Blue Coomassie staining. Note oligomerization of CPE-GST. Figure S2: Activity of GST-CPE and CPE treated with thrombin on Vero cells as monitored by entry of propidium iodide and cell viability MTT test as described in Figure 1. No significant difference was observed in the activity of CPE compared to GST-CPE.

## Abbreviations

CPE	*Clostridium perfringens* enterotoxin
Vero cells	African green monkey kidney epithelial cells
MTT	3-(4,5-dimethylthiazol-2-yl)-2,5-diphenyltetrazolium bromide
PI	propidium iodide
