# Supplementary material for: Clostridium perfringens Enterotoxin: The Toxin Forms Highly Cation-Selective Channels in Lipid Bilayers
Source: Toxins (Basel). 2018 Aug 22;10(9):341. doi: 10.3390/toxins10090341 (PMC6162509; doi:10.3390/toxins10090341)
Supplement: Supplementary file 1 [file toxins-10-00341-s001.pdf]

Supplementary Materials: *Clostridium perfringens* Enterotoxin : The Toxin Forms Highly Cation-Selective Channels in Lipid Bilayers

Roland Benz and Michel R. Popoff

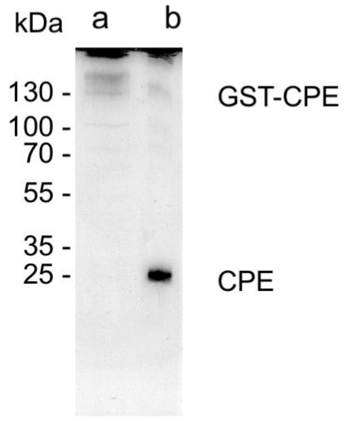

Figure S1. SDS-PAGE of GST-CPE and CPE. Lane a: GST-CPE, 3 µg; lane b, CPE (3 µg) after treatment of GST-CPE with biotinylated thrombin and removing by streptavidin agarose beads. Blue Coomassie staining. Note oligomerization of CPE-GST.

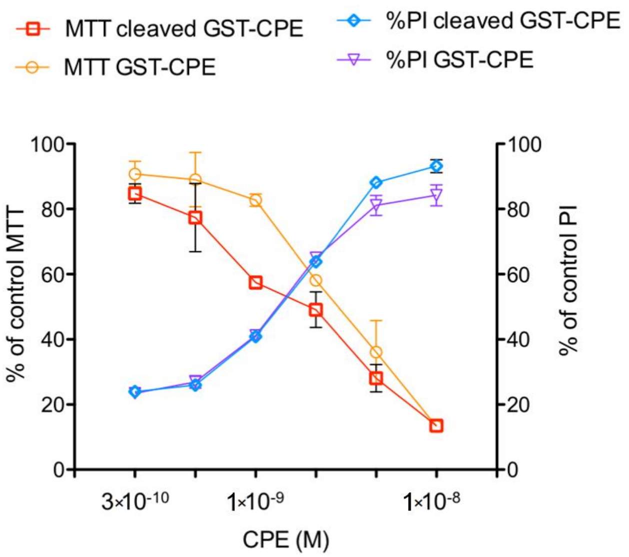

Figure S2. Activity of GST-CPE and CPE treated with thrombin on Vero cells as monitored by entry of propidium iodide and cell viability MTT test as described in Fig. 1. No significant difference was observed in the activity of CPE compared to GST-CPE.
